# Die Komplikationsrate nach Femurschaftfrakturen im Kindes- und Jugendalter in Abhängigkeit von Patientenfaktoren und Behandlungsmaßnahmen

**DOI:** 10.1007/s00104-021-01437-2

**Published:** 2021-06-16

**Authors:** S. Oberthür, S. Piatek, H. Krause, H. Rüther, P. J. Roch, A. Zoch, W. Lehmann, S. Sehmisch, M. R. Klauser

**Affiliations:** 1 Klinik für Unfallchirurgie, Orthopädie und Plastische Chirurgie, Universitätsmedizin Göttingen, Robert-Koch-Straße 40, 37075 Göttingen, Deutschland; 2 Universitätsklinik für Unfallchirurgie, Otto-von-Guericke Universität Magdeburg, Magdeburg, Deutschland; 3 Bereich Kinderchirurgie und Kindertraumatologie, Otto-von-Guericke-Universität Magdeburg, Magdeburg, Deutschland; 4 Abteilung Volkswirtschaftslehre, Universität Mannheim, Mannheim, Deutschland

**Keywords:** Elastisch stabile intramedulläre Nagelung (ESIN), Fixateur externe, Konservative Therapie, Komplikationen, Limitationen, Elastic stable intramedullary nailing (ESIN), External fixation, Conservative treatment, Complications, Limitations

## Abstract

**Hintergrund:**

Die Behandlung der Femurschaftfrakturen bei Kindern war in den letzten Jahren einem zunehmenden Wandel unterzogen. Die früher dominierenden Therapieverfahren wurden durch minimal-invasive Techniken (z. B. elastisch stabile intramedulläre Nagelung [ESIN]) weitgehend abgelöst. Ziel der vorliegenden Studie war der Vergleich der Komplikationsraten in Abhängigkeit sowohl von Patientenfaktoren als auch von verschiedenen Behandlungsverfahren.

**Material und Methoden:**

Bei der vorliegenden Arbeit handelt es sich um eine retrospektive röntgenmorphometrische Datenauswertung. Es wurden die Patientenakten und Röntgenbilder von 101 Kindern, die an zwei Level-I-Traumazentren behandelt wurden, ausgewertet.

**Ergebnisse:**

In 19 % der Fälle wurde eine konservative Therapie durchgeführt. Bei den operativen Verfahren dominierte die ESIN-Technik (*n* = 60). Revisionspflichtige Komplikationen fanden sich nach konservativer Therapie bei ca. 10 % der Kinder. Bei den operativ behandelten Kindern musste in über 6 % der Fälle eine Revisionsoperation durchgeführt werden. Die ESIN-Stabilisierung zeigte bei den operativen Verfahren mit ca. 3 % die niedrigste Revisionsrate. Wurden im Verhältnis zum Markraumdurchmesser zu dünne ESIN-Drähte verwendet, so zeigte sich eine um 30 % höhere Komplikationswahrscheinlichkeit. Bei Kindern unter 3 Jahren und Adoleszenten war die Komplikationswahrscheinlichkeit erhöht.

**Diskussion:**

In der durchgeführten Studie zeigt sich ein moderates Komplikationsrisiko bei der Behandlung von Femurschaftfrakturen bei Kindern. Das Komplikationsrisiko nach Fixateur-externe-Anlage und konservativer Behandlung war in dieser Studie am höchsten. Die ESIN-Technik zeigt insgesamt das geringste Komplikationsrisiko. Die vorliegende Arbeit konnte die bekannten Limitationen der ESIN-Technik in Abhängigkeit von Alter und Gewicht bestätigen.

## Abstract (en)

**Background:**

The treatment of pediatric femoral shaft fractures has undergone an increasing change in recent years. The previously predominant treatment procedures were extensively replaced by minimally invasive techniques (e.g. elastic stable intramedullary nailing, ESIN). The aim of this study was the comparison of complication rates depending on patient factors as well as various treatment procedures.

**Material and methods:**

This study involved a retrospective X‑ray morphometric evaluation of data. The patient files and X‑rays of 101 children who were treated at 2 level I trauma centers were analyzed.

**Results:**

Conservative treatment was carried out in 19% of the cases. Among the surgical procedures the ESIN technique was predominant (*n* = 60). Complications that needed revision occurred in 10% of the children after conservative treatment. Revision surgery had to be carried out in more than 6% of the cases in children who were surgically treated. Among the surgical procedures ESIN stabilization demonstrated the lowest revision rate with only 3%. Children under three years and adolescents had a higher risk for developing complications. If the ESIN wires used were too thin in relation to the diameter of the medullary cavity there was an increased probability of complications of around 30%.

**Conclusion:**

This study revealed a moderate risk of complications in the treatment of femoral shaft fractures in children. The risk of complications after external fixation and conservative treatment was the highest in this study. Overall, the ESIN technique showed the lowest risk of complications. The results of this study could confirm the known limitations of the ESIN technique depending on age and body weight.

## Hintergrund

Die Femurschaftfraktur hat einen Anteil von bis zu 1,5 % bei kindlichen Frakturen und ist eine häufige Fraktur der unteren Extremität bei Kindern [1, 2]. Die Inzidenz wird mit 11–26/100.000 Kinder/Jahr angegeben [3, 4].

In den vergangenen Jahren hat sich die Therapie der Femurschaftfraktur bei Kindern und Heranwachsenden stark gewandelt. Während früher gerade bei jungen Kindern die konservative Therapie im Vordergrund stand, wird zunehmend die operative Behandlung bevorzugt. Zu den konservativen Therapieoptionen zählen die Behandlung mit Oberschenkel-Cast, der Becken-Bein-Gips und die Overhead-Extension. Bei der konservativen Therapie entfallen die Risiken invasiver Methoden, jedoch muss sich der Patient einer mehrwöchigen Immobilisation, ggf. einem längeren Krankenhausaufenthalt und wiederholten Röntgenuntersuchungen unterziehen. Nach Marzi et al. sollten diaphysäre Frakturen bei unter 5‑Jährigen 1 bis 3 Wochen, bei 5‑ bis 10-Jährigen 4 bis 5 Wochen und bei Kindern über 10 Jahren 4 bis 6 Wochen ruhiggestellt werden [5]. Komplikationen einer nicht adäquaten konservativen Therapie können u. a. Druckulzera, Nervenschäden, sekundäre Dislokationen und bleibende Fehlstellungen sein [5, 6].

Operativ stehen extramedulläre (Fixateur externe, Platte, Schrauben) und intramedulläre (elastisch stabile intramedulläre Nagelung [ESIN], Verriegelungsnägel) Verfahren zur Verfügung. Am häufigsten werden Femurfrakturen bei Kindern im Alter von 3 bis 15 Jahren heute mittels ESIN versorgt. Bei der ESIN-Technik wird die Fraktur durch die Aufspannung von zwei vorgebogenen Titandrähten im Markraum stabilisiert. Vorteile der ESIN-Osteosynthese sind die Minimalinvasivität, die indirekte Frakturheilung sowie die sofort mögliche Teilbelastung ohne weitere Ruhigstellung.

Während die Leitlinie der Deutschen Gesellschaft für Kinderchirurgie (S1-AWMF[Arbeitsgemeinschaft der Wissenschaftlichen Medizinischen Fachgesellschaften]-Leitlinie 006/016 von 09/2014 – keine aktuelle Gültigkeit) die ESIN-Therapie bei Femurschaftfrakturen erst ab dem 3. Lebensjahr empfiehlt, wird diese in der Realität in Deutschland bereits in 50 % der Fälle auch bei jüngeren Kindern angewendet [7, 8].

Neben den minimal-invasiven Verfahren steht als Alternative bei adoleszenten und/oder adipösen Kindern sowie bei langen Spiral- oder Mehrfragmentfrakturen die minimal-invasive perkutane Plattenosteosynthese (MiPo-Technik) zur Verfügung [9]. In seltenen Fällen, bei drittgradig offenen Frakturen, polytraumatisierten Patienten oder sehr instabilen Spiral- oder Mehrfragmentfrakturen kann alternativ ein Fixateur externe ggf. auch in Kombination mit ESIN verwendet werden [5, 10].

Die Literatur gibt sehr unterschiedliche Komplikationsraten bei der Behandlung von Femurschaftfrakturen bei Kindern und Jugendlichen von 6–50 % an. Die Analyse der Literatur zeigt weiterhin eine Kontroverse bezüglich der zu wählenden Therapieform bei Kindern verschiedenen Alters und Gewichts. Um diese Frage näher zu untersuchen, wurde in dieser retrospektiven Studie an zwei Level-I-Traumazentren das Komplikationsspektrum mit den entsprechenden Risikofaktoren in Abhängigkeit der Behandlungsform und -technik bei Femurschaftfrakturen im Kindes- und Jugendalter analysiert.

## Material und Methoden

In einer retrospektiven röntgenmorphometrischen Untersuchung wurden an zwei Level-I-Traumazentren der Behandlungsablauf und die Komplikationen bei der Behandlung von Oberschenkelschaftfrakturen über je 6 Jahre analysiert. Einbezogen wurden Kinder im Alter von 0 bis 16 Jahren. Neben diesen Daten wurden das Patientenalter, die Größe, das Gewicht, der Frakturtyp und die gewählte Therapie erfasst. Da sich die vorliegende Studie insbesondere mit dem Komplikationsspektrum der ESIN-Therapie auseinandersetzt, wurden die technischen Parameter (ESIN-Dicke) und die postoperativen Komplikationen ermittelt und ein möglicher Zusammenhang untersucht.

**Figure 1:**
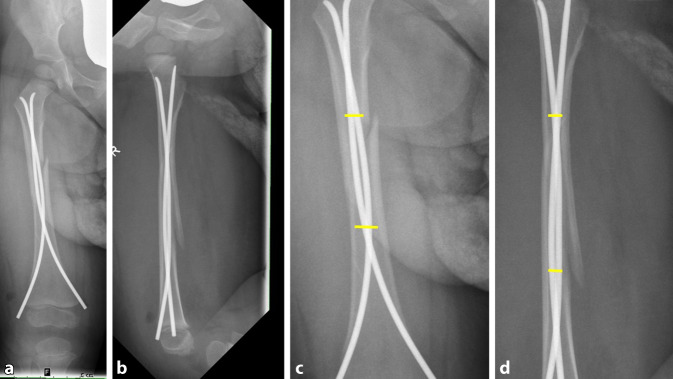

Hierfür wurden nach ESIN-Implantation die postoperativen Röntgenbilder ausgewertet. Dabei wurde der Durchmesser des Markraums proximal und distal des wesentlichen Frakturbereichs in Millimeter in beiden radiologischen Ebenen (a.p. und seitlich) gemessen und der Mittelwert bestimmt. Anschließend wurde das Verhältnis von Markraumdurchmesser zur Dicke des ESIN berechnet (Mittelwert proximaler und distaler Markraumdurchmesser : 2‑mal ESIN-Dicke; Abb. 1).

Die statistische Analyse und Darstellung der Resultate wurde mit Stata/SE 13.1 (StataCorp LP, College Station, USA) durchgeführt. Das Signifikanzniveau lag bei *p* < 0,05. Für die Daten wurden t‑Tests, logistische, multivariate und Kernel-Regressionen berechnet. Da es sich bei den vorliegenden Ergebnissen nicht um lineare Zusammenhänge handelt, wurde die Kernel-Regression verwendet. Die multivariable Testung wurde vorgenommen, um verschiedene Parameter in Verbindung zu setzen.

## Ergebnisse

**Figure 2:**
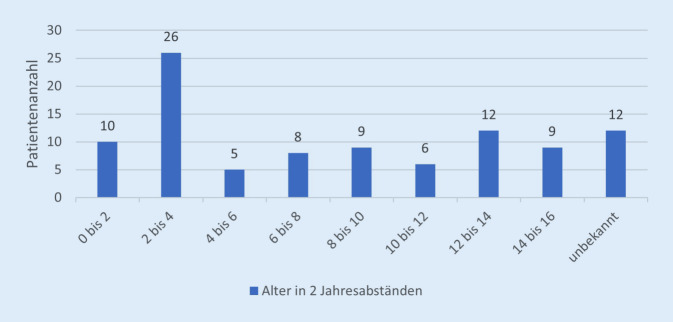

Im Beobachtungszeitraum wurden an beiden Zentren insgesamt 101 Patienten mit einer Femurschaftfraktur in die Studie eingeschlossen. Das Patientenkollektiv setzte sich aus 68 Jungen (67,3 %) und 33 Mädchen (32,7 %) zusammen. In Tab. 1 sind die Daten der untersuchten Kohorte zusammengefasst. Das Durchschnittsalter aller Patienten lag bei 7 Jahren (0–15 Jahre). Der Median in unserem Patientenklientel lag bei 6,3 Jahren (Minimum 3 Monate, Maximum 15 Jahre). Ein Drittel aller Frakturen ereignete sich im Alter von 2 bis 4 Jahren (Abb. 2).

**Table 1:** 

Patientendaten	Anzahl
*Patientenzahl*	101
Jungen	68
Mädchen	33
*Frakturform*	
Spiralfraktur	32
Querfraktur	15
Schrägfraktur	15
Mehrfragmentfraktur	8
Keine Angabe	31
*Therapie*	
Konservativ	19
Operativ	77
Sonstige	1
*Operationsverfahren*	
ESIN	60
Plattenosteosynthese	11
Fixateur externe	5
Marknagel	1

*ESIN* elastisch stabile intramedulläre Nagelung

Die Spiralfraktur des Femurs war mit 31,7 % (*n* = 32) die häufigste Frakturform. Mit gleicher Häufigkeit folgten Quer- und die Schrägfrakturen (je 14,9 %). In 8 Fällen wurden Mehrfragmentfrakturen diagnostiziert (Tab. 1).

In 97 (96 %) Fällen konnten sämtliche Auswertungsparameter retrospektiv erhoben werden. Nur diese Patienten wurden in die Analyse eingeschlossen. Eine konservative Therapie wurde bei 19 Kindern (19,6 %) durchgeführt. Das Durchschnittsalter in dieser Gruppe betrug 3,1 Jahre. Als Techniken der nicht operativen Behandlung erfolgten altersabhängig eine Cast-Behandlung oder eine Extensionsbehandlung. Bei 77 Kindern (79,4 %) wurde operativ therapiert. Die operativ behandelten Patienten hatten im Durchschnitt ein Alter von 7,2 Jahren. Bei einem Patienten wurde eine Knochenkürretage bei einer pathologischen Fraktur durchgeführt – dieser wurde als Sonderfall nicht in die weitere Auswertung einbezogen.

**Figure 3:**
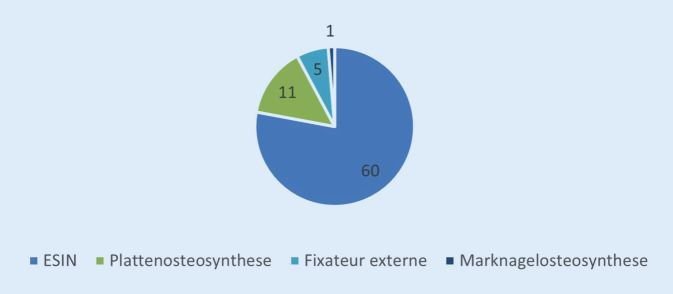

Bei der operativen Therapie wurde in 77,9 % (*n* = 60) der Fälle eine Stabilisierung mittels ESIN durchgeführt (Durchschnittsalter 5,8 Jahre). Bei allen Patienten wurden die ESIN in aszendierender Technik eingebracht. Zudem wurden jeweils zwei ESIN der gleichen Materialdicke verwendet. Bei 14,3 % (*n* = 11) der operierten Kinder wurde eine minimal-invasive Plattenosteosynthese (Durchschnittsalter 12,6 Jahre), bei 6,5 % (*n* = 5) eine Therapie mit Fixateur externe (Durchschnittsalter 9,9 Jahre) und bei 1,3 % (*n* = 1 mit 15,5 Jahren) eine Marknagelosteosynthese mit einem Adoleszentennagel durchgeführt (Tab. 2; Abb. 3). Bei 75 % aller Operationen konnte eine geschlossene Reposition der Fraktur erreicht werden. Der Krankenhausaufenthalt betrug durchschnittlich 12 Tage (3–34 Tage).

**Table 2:** 

Frakturtyp	Art der Versorgung				
	ESIN	Platte	Fixateur externe	Nagel	Total
Spiralfraktur	23	3	1	0	27
Querfraktur	10	4	0	0	14
Schrägfraktur	10	2	1	0	13
Mehrfragmentfraktur	2	2	2	1	7
Femurschaftfraktur o.n.A.	15	0	1	0	16
*Total*	*60*	*11*	*5*	*1*	*77*

*ESIN* elastisch stabile intramedulläre Nagelung, *o.n.A.* ohne nähere Angaben

**Table 3:** 

Variablen	Komplikationen
Alter bei Operation > 3 und < 8,5 Jahre	−0,274* (0,0507)
Alter bei Operation ≥8,5 u. <12	0,0767 (0,627)
Alter bei Operation ≥12 u. <14	0,424* (0,0560)
Alter bei Operation ≥14	−0,235 (0,412)
Verhältnis ≥1,5 u. <2	0,209 (0,136)
Verhältnis ≥2	0,0268 (0,845)
	0,221* (0,0750)
Beobachtungen	49
R^2^	0,304

*p*-Werte in Klammern: ***p < 0,01, **p < 0,05, *p < 0,1

In 78,2 % aller Fälle wurden keine Komplikationen nach konservativer bzw. operativer Behandlung beobachtet. Revisionspflichtige Komplikationen fanden sich im Gesamtkollektiv bei 7,2 % der Kinder. Nach einer konservativen Behandlung wurde bei 10,5 % der Kinder ein relevanter Repositionsverlust beobachtet, der die altersspezifischen Korrekturgrenzen überstieg und anschließend operativ korrigiert werden musste. Bei den operativ therapierten Frakturen musste in 6,5 % eine Revisionsoperationen durchgeführt werden (2-mal, 2‑mal Fixateur externe, 1‑mal Platte). Bei 2 Kindern erfolgte die Revision bei einer postoperativen Varusfehlstellung (1-mal ESIN, 1‑mal Platte) und bei einem Kind aufgrund eines Teleskopierens nach ESIN-Stabilisierung. Während bei 66 % (zwei Drittel) der Kinder mit Fixateur externe bei einem Repositionsverlust ein Verfahrenswechsel notwendig wurde, betrug die Revisionsrate nach ESIN-Therapie insgesamt 3,3 % (2/60). Bei den Frakturen, die zu einem Versagen der ESIN führten, handelte es sich ausschließlich um Schrägfrakturen.

Leichte Einschränkungen ohne Revisionsbedarf traten bei 21,8 % (*n* = 22) aller behandelten Kinder auf. Von diesen hatten 68,2 % (15/22) eine ESIN-Stabilisierung erhalten. Es wurden Hämatome, Wundheilungsstörungen, hypertrophe Narben und temporäre Funktionseinschränkungen an Hüft- oder Kniegelenk beobachtet.

**Figure 4:**
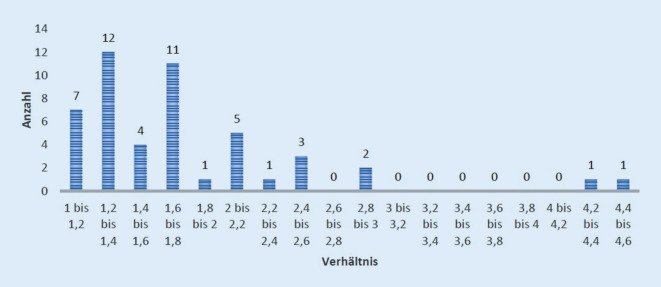

Bei 54 (87,1 %) Patienten konnten nach ESIN-Therapie die postoperativen Röntgenbilder ausgewertet werden. Bei der Berechnung des Verhältnisses zwischen Markraumdurchmesser zu beiden ESIN zeigte sich am häufigsten ein Wert von 1,2 bis 1,4 (25 %; Abb. 4). In 22,9 % der Fälle wurden dünnere ESIN eingebracht, sodass sich ein Verhältnis von 1,6 bis 1,8 ergab. Der kleinste Quotient betrug 1,0 und konnte bei 2 Patienten beobachtet werden. Bei diesen wurde der Markraum durch beide ESIN vollständig ausgefüllt. Der größte Quotient wurde mit 4,52 in einem Fall ermittelt. Insgesamt ergab sich im Durchschnitt ein Verhältnis von 1,77 – die ESIN füllten hier mehr als die Hälfte des Markraums aus.

**Figure 5:**
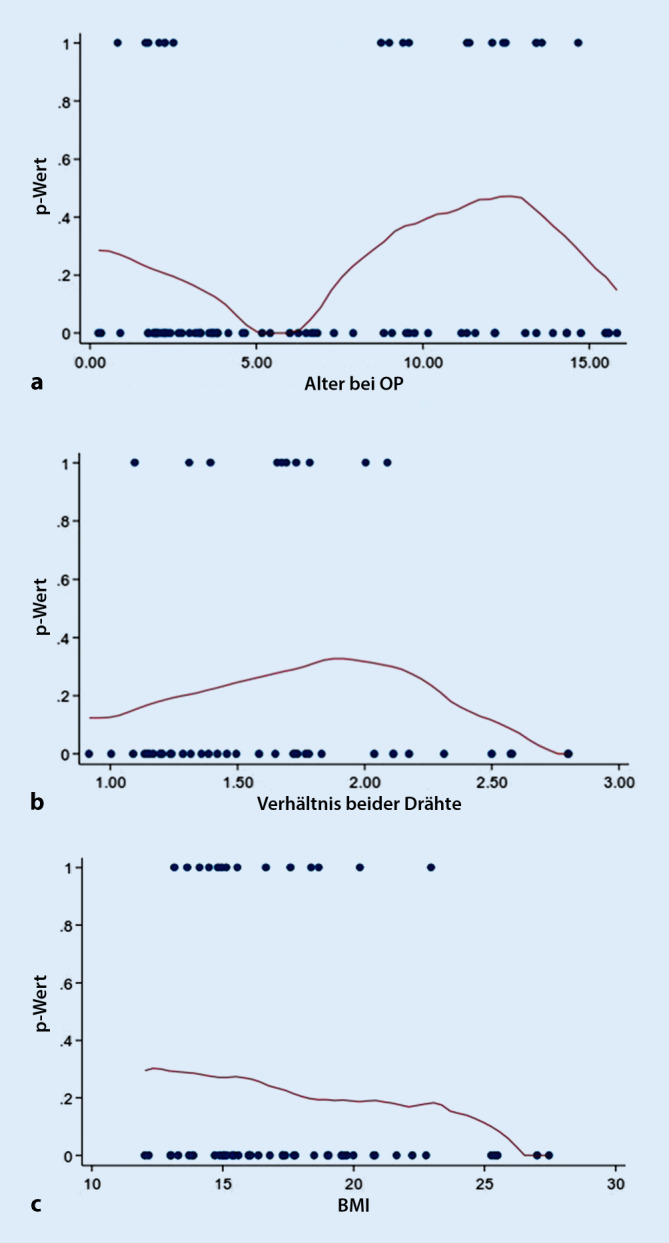

Bei der Untersuchung dieses berechneten Quotienten im Hinblick auf postoperative Komplikationen konnte ein interessanter Zusammenhang nachgewiesen werden. In der Untersuchung zeigte sich, dass ein Quotient (Markraumdurchmesser: 2‑mal ESIN-Drahtdicke) zwischen 1,5 bis 2 zu einem um 30 % signifikant höheren Komplikationsrisiko im Vergleich zu einem Verhältnis von kleiner 1,5 führt. Bei einem Quotienten = 1 scheint das Komplikationsrisiko unter 20 % zu liegen (Abb. 4 und 5). Bei einem Verhältnis von > 2 konnte aufgrund der geringen Fallzahl kein signifikanter Zusammenhang mehr nachgewiesen werden (Abb. 5; Tab. 3).

Betrachtet man den Einflussfaktor „Alter“ im Hinblick auf postoperative Komplikationen, so konnte ebenfalls ein Zusammenhang beobachtet werden. Das Risiko, Komplikationen zu erleiden, sinkt von Geburt bis zum 5. Lebensjahr kontinuierlich. Danach steigt es an und erreicht um das 12./13. Lebensjahr sein Maximum. Anschließend nimmt es wieder ab (Abb. 5; Tab. 3). Es konnte ebenfalls gezeigt werden, dass Patienten zwischen 3 und 8,5 Jahren das geringste Risiko für Komplikationen aufweisen. Ihr Risiko war statistisch signifikant um 28 % (*p* = 0,015) im Vergleich zu Kindern jünger als 3 Jahre reduziert. Die Gruppe der 12- bis 13-jährigen Kinder hatte ein höheres Risiko für Komplikationen (26,5 %, *p* < 0,066) als die Kinder unter dem 3. Lebensjahr.

Diese Effekte sind robust für das Multivariablenmodell, wenn man das Verhältnis der ESIN-Dicke (Verhältnis 1,5–2,0) einbezieht. Für das Komplikationsrisiko spielen demnach neben den Altersgruppen 3 bis 8,5 und 12 bis 13 Jahre auch das Verhältnis Markraumdurchmesser/ESIN-Dicke (1,5–2) eine signifikante Rolle. Diese beiden Variablen erklären 30 % der Komplikationen in dieser Untersuchung [11].

Im t‑Test zeigte sich zudem, dass eine offene Reposition (33 %) zu einem signifikant (*p* = 0,010) höheren Komplikationsrisiko im Vergleich zu einer geschlossenen Reposition (20 %) führt.

## Diskussion

Die Therapie der Femurschaftfraktur von Kindern und Heranwachsenden hat sich in den letzten Jahren von der einstigen konservativen Domäne zur operativen Behandlung verschoben. Die Ende der 1970er-Jahre entwickelten und heute favorisierten minimal-invasiven Behandlungsoptionen (ESIN) scheinen in Abhängigkeit von Patientenfaktoren (Alter, Body-Mass-Index) der konservativen Therapie überlegen zu sein [7, 8, 12]. Bei diesem Verfahren ist die korrekte Operationstechnik entscheidend. In der Literatur wird v. a. auf eine korrekte Nageldicke sowie ein ausreichendes Vorbiegen der Nägel hingewiesen [9]. Außerdem wird das Gewicht des Kindes als relevanter Risikofaktor beschrieben. Mögliche Komplikationen der ESIN-Therapie sind u. a. Bewegungseinschränkungen der Kniegelenksflexion, Wundheilungsstörungen, Instabilität bzw. Teleskopieren der Nägel mit sekundärer Dislokation und in der Folge dann Wachstumsstörungen mit Beinlängendifferenzen [4, 13]. In der Literatur wird kritisch diskutiert, ab bzw. bis zu welchem Alter oder Gewicht und bei welchem Frakturtyp welches Therapieregime gewählt werden sollte [7]. Um sich dieser Frage zu nähern, wurde in einer retrospektiven Studie das Komplikationsspektrum nach Behandlung einer Femurschaftfraktur bei Kindern analysiert.

Die Ergebnisse dieser Untersuchung zeigen, dass Femurschaftfrakturen bei Kindern und Jugendlichen in den meisten Fällen komplikationslos (78,2 %) und mit guten Ergebnissen behandelt werden können. Über 20 % der Verletzten berichteten nach der operativen Behandlung dennoch über Einschränkungen und Beschwerden. Die Behandlung wurde entsprechend den aktuell gültigen Behandlungsempfehlungen mit ESIN (68,1 %) durchgeführt. Die Autoren der Studie unterschieden zwischen behandlungsabhängigen Einschränkungen bzw. Komplikationen, die keiner Revisionsoperation bedurften, und Komplikationen, die zu einer Revision führten. Einschränkungen ohne Notwendigkeit einer chirurgischen Revision zeigten sich mit 23 % signifikant häufiger bei operativ behandelten Kindern (15 % nach konservativer Therapie). Zu den 23 % Komplikationen in der Gruppe der operativ Behandelten zählen häufig temporäre Beschwerden, wie z. B. eine eingeschränkte Kniegelenksbeweglichkeit, die durch Irritationen der ESIN am Traktus gelegentlich zu beobachten ist.

Revisionspflichtige und damit relevante Komplikationen zeigten immerhin 6,5 % der operativ behandelten Kinder. Die Revisionsrate nach ESIN-Osteosynthese lag hier mit 3,2 % deutlich unter diesem Wert. Im Gegensatz dazu mussten 10,5 % der konservativ behandelten Kinder im Verlauf operativ revidiert werden. Zu übereinstimmenden Ergebnissen kamen die Untersuchungen von Poolman et al. [4] und Buechsenschuetz et al. [14]. Poolmann et al. analysierten in einer Metaanalyse 33 Studien mit insgesamt 2422 Patienten mit Femurschaftfraktur und stellten fest, dass die operative Therapie und insbesondere die ESIN-Therapie im Vergleich zur konservativen Therapie insgesamt komplikationsärmer sei [4]. Auch Buechsenschuetz et al. konnten in ihren Untersuchungen zeigen, dass die konservativ behandelten Femurschaftfrakturen verglichen mit der ESIN-Gruppe eine doppelt so hohe Komplikationsrate (44 % vs. 22 %) hatten [14]. Die Komplikationsraten in der vorliegenden Studie mit weniger Patienten zeigt eine geringere Komplikationsrate. Jedoch sind die Ergebnisse der Studie von Buechsenschuetz et al. deutlich älter. Ursächlich für die besseren Ergebnisse könnten technische Innovationen und Erfahrungen seit den Daten von Buechsenschuetz et al. [14] sein.

In den vorliegenden Untersuchungen hatten sich innerhalb der operativen Verfahren deutliche Unterschiede bei der Komplikationsrate gezeigt. Die höchste Revisionsrate wurde nach Behandlung im Fixateur externe beobachtet. Maier et al. [13] kamen in ihren Untersuchungen zu ähnlichen Ergebnissen. Die Komplikationsrate lag bei 14 % bei konservativer Therapie, 6 % bei der ESIN-Behandlung und 44 % bei der Behandlung mit Fixateur externe. Insgesamt zeigte demnach die konservative Therapie verglichen mit allen operativen Ansätzen die besseren Ergebnisse [15]. Betrachtet man die verschiedenen operativen Therapieverfahren, so war die ESIN-Versorgung am komplikationsärmsten.

Die Untersuchungen von Ramseier et al. [16] analysierten die Revisionsrate bei 104 operativ versorgten Kindern mit Oberschenkelschaftfrakturen – auch hier mussten am häufigsten Kinder nach Fixateur-externe-Versorgung revidiert werden (52 % – 16/33; [16]). Die Revisionsrate nach ESIN-Implantation hingegen betrug 8 %.

In der vorliegenden Studie zeigt sich ein deutlicher Anstieg der Komplikationsrate bei ESIN-Behandlung, wenn zu dünne ESIN verwendet werden. Bei der Untersuchung der ESIN-Dicke im Verhältnis zum Markraumdurchmesser hat sich gezeigt, dass ein Quotient (Markraumdurchmesser: 2‑mal ESIN-Drahtdicke) zwischen 1,5 und 2 ein 30 % höheres Komplikationsrisiko bedeutet verglichen mit einem Verhältnis < 1,5. Bei einem Verhältnis von 1 bis 1,5 ist der Markraum 66–100 % ausgefüllt. Hier zeigte sich in der vorliegenden Studie die geringste Komplikationsrate. Dies korreliert mit der Herstellerempfehlung, möglichst eine Drahtdicke von mindestens 40 % der Markraumbreite zu wählen – bei zwei Nägeln 80 % entsprechend. Auch die Studie von Flinck et al. [17] zeigte ähnliche Ergebnisse. Hier wurden am Frakturmodell ESIN sowie ein Marknagel getestet. Je weiter der Markraum durch die Implantate ausgefüllt war, desto stabiler erwies sich die Fraktur [17]. Auch Lascombes et al. [18] stellten fest, dass das Outcome bei ESIN-Osteosynthesen signifikant durch die gewählte Nageldicke beeinflusst wird. Die Nageldicke sollte mehr als 40 % des Markraumdurchmessers betragen. Bei einem Markraumdurchmesser von mehr als 10 mm empfehlen sie die Verwendung einer alternativen Operationstechnik [18]. In der Arbeit von Nielsen et al. [18] konnte hingegen kein Zusammenhang zwischen der Stärke der ESIN und dem Komplikationsspektrum nachgewiesen werden [19]. Jedoch wurde in dieser Studie mit 66 Patienten der Markraum durchschnittlich zu 76,3 % ausgefüllt [19]. Nur bei 13,6 % der Kinder aus der Arbeit von Nielsen et al. wurde ein ESIN-Durchmesser von unter 60 % im Verhältnis zum Markraum verwendet [19]. Somit stützen diese Ergebnisse die Schlussfolgerungen der vorliegenden Arbeit. Obwohl ein Verhältnis von 1,0 bis 1,5 biomechanisch die höchste Stabilität verspricht, besteht bei einem Quotienten nahe 1,0 die Gefahr der Schaftsprengung bzw. einer erschwerten Implantatentfernung.

Hervorzuheben ist, dass bei der ESIN-Osteosynthese das Komplikationsrisiko ansteigt, wenn der unausgefüllte Markraum mehr als 25 % beträgt. Entscheidend für eine erfolgreiche ESIN-Therapie ist daher die korrekte Operationstechnik [9]. Zur biomechanischen Optimierung von ESIN-Osteosynthesen stehen zusätzlich Hilfsmittel wie „end caps“ oder verriegelbare ESIN zur Verfügung, um einer sekundären Dislokation vorzubeugen. Die indikationsgerechte Verwendung dieser Hilfsmittel, könnte die Ergebnisse weiter verbessern.

Betrachtet man das Alter als Risikofaktor für Komplikationen, so hat sich in der aktuellen Studie gezeigt, dass die Gruppe der Kinder zwischen 3 und 8,5 Jahren das geringste Komplikationsrisiko hat, gefolgt von Kindern unter 3 Jahren. Dieser Unterschied ist statistisch signifikant. Die Leitlinie der Deutschen Gesellschaft für Kinderchirurgie empfiehlt bis zum 3. Lebensjahr konservativ zu therapieren [8]. Die Ergebnisse dieser Studie unterstützen diese Empfehlung – die operative Therapie bei unter 3‑Jährigen birgt signifikant mehr Komplikationen als die operative Versorgung bei Kindern zwischen 3 und 8,5 Jahren. Möglicherweise gibt es in dieser Gruppe andere anatomische Gegebenheiten als in höherem Alter. Seit den 1990er-Jahren wurde in verschiedenen Untersuchungen gezeigt, dass das Frakturrisiko im Alter von 12 bis 13 Jahren stark ansteigt. Postuliert wurde, dass dies im Zusammenhang mit den Umbauvorgängen des Knochens während des letzten Wachstumsschubes steht [20–22]. Durch Bonjour und Fournier et al. wurde ein zeitliches Ungleichgewicht bei der Zunahme von Knochenlänge im Verhältnis zur Knochenmasse beschrieben [21, 22]. Die größte Differenz entsteht mit dem pubertären Wachstumsschub. Vermutet wird, dass aufgrund dieses relativen Defizits des Knochenmineralsalzgehaltes eine erhöhte Fragilität für den jugendlichen Knochen besteht [21, 22].

In dieser Arbeit war demzufolge für die 12- bis 13-jährigen Kinder das Komplikationsrisiko signifikant am höchsten. In dieser Altersspanne sollte deshalb die Indikation für ESIN-Therapien besonders geprüft werden, da diese eventuell keine ausreichende Stabilität in dieser pubertären Umbauphase bieten könnten. So zeigte sich in der Studie von Moroz ein 5‑fach erhöhtes Komplikationsrisiko bei Kindern über 49 kg [13]. Bei schwereren Kindern (> 50 kg) oder älteren Kindern wird die Verwendung des Adoleszentenfemurnagels empfohlen [5]. Reynolds et al. verglichen in ihrer Studie die Komplikationen nach ESIN bzw. Adoleszentenmarknagelimplantation [23]. Sie konnten zeigen, dass sich die Komplikationsrate nicht signifikant unterschied und in der Gruppe der Adoleszentenmarknägel keine schweren Komplikationen auftraten. Außerdem belasteten die Kinder mit Marknagel ihr verletztes Bein deutlich schneller. Martus [15] untersuchte in einer Metaanalyse das Outcome von Heranwachsenden, die mit dem Adoleszentenmarknagel behandelt wurden. Es zeigten sich gute Therapieergebnisse, sodass man diese Technik bei großen und schweren Kindern im kritischen Alter von 12 bis 13 Jahren verwenden sollte [24].

Die Grenzen der Aussagekraft des Manuskriptes liegen in dem retrospektiven Studiendesign, dem heterogenen Patientenklientel sowie der Patientenanzahl. Zukünftige Studie sollten diese Fragestellungen in Multicenter‑/Registerstudien adressieren.

## Fazit für die Praxis

- Die Femurschaftfraktur ist eine relevante Verletzung im Kindesalter. Derzeit ist bei vielen Kindern die operative Behandlung mittels ESIN-Osteosynthese zum Goldstandard geworden. Insgesamt haben die Ergebnisse dieser Studie gezeigt, dass die operative Therapie der Femurschaftfrakturen im Kindes- und Jugendalter eine sichere Behandlungsoption darstellt – Grundlage der effektiven Therapie ist jedoch die richtige Indikationsstellung für das jeweilige Behandlungsverfahren.
- Die ESIN-Osteosynthese ist ab dem 3. Lebensjahr zu empfehlen und zeigte signifikant das niedrigstes Komplikationsrisiko für Kinder im Alter zwischen 3 und 9 Jahren. Zudem konnte anhand der Ergebnisse der Arbeit das ideale Verhältnis von ESIN-Durchmesser zu Markraum (> 1 bis 1,5) ermittelt werden, um das Risiko für Komplikationen zu minimieren.

## References

[1] von Laer L, Kraus R, Linhart WE (2012). Frakturen und Luxationen im Wachstumsalter.

[2] Dietz H-G, Schlickewei W (2011). Femurschaftfrakturen im Kindesalter (Femoral shaft fractures in children). Unfallchirurg.

[3] Hedström EM, Svensson O, Bergström U (2010). Epidemiology of fractures in children and adolescents. Acta Orthop.

[4] Poolman RW, Kocher MS, Bhandari M (2006). Pediatric femoral fractures: a systematic review of 2422 cases. J Orthop Trauma.

[5] Marzi I (2010). Kindertraumatologie.

[6] Kraus R, von Laer L (2007). Technische Komplikationen bei der Versorgung von Frakturen. Das verletzte Kind.

[7] Strohm PC, Schmittenbecher PP (2015). Femurschaftfrakturen bei Kindern unter 3 Jahren. Aktueller Behandlungsstandard (Femoral shaft fractures in children under 3 years old. Current treatment standard). Unfallchirurg.

[8] Gresing T, Illing P (2010) Femurschaftfraktur – AWMF Leitlinie der Deutschen Gesellschaft für Kinderchirurgie.: (AWMF online). http://www.awmf.org/leitlinien/detail/ll/006-016.html

[9] Worel AM, Slongo T, Marzi I (2010). Behandlungsprinzipien. Kindertraumatologie.

[10] von Laer L (2007). Das verletzte Kind.

[11] Klauser MR (2019). Komplikationen und Komplikationsrisiken bei der Versorgung kindlicher Femurschaftfrakturen: Statistische Analyse an den Traumazentren der Universitätsmedizin.

[12] Slongo T (2015). Was tun, wenn die elastisch-stabile intramedulläre Nagelung (ESIN) an ihre Grenzen stößt?. Trauma Berufskrankh.

[13] Moroz LA, Launay F, Kocher MS (2006). Titanium elastic nailing of fractures of the femur in children. Predictors of complications and poor outcome. J Bone Joint Surg Br.

[14] Buechsenschuetz KE, Mehlman CT, Shaw KJ (2002). Femoral shaft fractures in children: traction and casting versus elastic stable intramedullary nailing. J Trauma.

[15] Maier M, Maier-Heidkamp P, Lehnert M (2003). Ausheilungsergebnisse konservativ und operativ versorgter kindlicher Femurfrakturen (Results of femoral shaft fractures in childhood in relation to different treatment modalities). Unfallchirurg.

[16] Ramseier LE, Janicki JA, Weir S (2010). Femoral fractures in adolescents: a comparison of four methods of fixation. J Bone Joint Surg Am.

[17] Flinck M, von Heideken J, Janarv P-M (2015). Biomechanical comparison of semi-rigid pediatric locking nail versus titanium elastic nails in a femur fracture model. J Child Orthop.

[18] Lascombes P, Huber H, Fay R (2013). Flexible intramedullary nailing in children: nail to medullary canal diameters optimal ratio. J Pediatr Orthop.

[19] Nielsen E, Bonsu N, Andras LM (2018). The effect of canal fill on paediatric femur fractures treated with titanium elastic nails. J Child Orthop.

[20] Parfitt AM (1994). The two faces of growth: benefits and risks to bone integrity. Osteoporos Int.

[21] Bonjour J-P, Chevalley T (2014). Pubertal timing, bone acquisition, and risk of fracture throughout life. Endocr Rev.

[22] Fournier PE, Rizzoli R, Slosman DO (1997). Asynchrony between the rates of standing height gain and bone mass accumulation during puberty. Osteoporos Int.

[23] Reynolds RAK, Legakis JE, Thomas R (2012). Intramedullary nails for pediatric diaphyseal femur fractures in older, heavier children: early results. J Child Orthop.

[24] Martus JE (2016). Rigid intramedullary nailing of femoral shaft fractures for patients age 12 and younger: indications and technique. J Pediatr Orthop.

